# Exome sequencing in mostly consanguineous Arab families with neurologic disease provides a high potential molecular diagnosis rate

**DOI:** 10.1186/s12920-016-0208-3

**Published:** 2016-07-19

**Authors:** Wu-Lin Charng, Ender Karaca, Zeynep Coban Akdemir, Tomasz Gambin, Mehmed M. Atik, Shen Gu, Jennifer E. Posey, Shalini N. Jhangiani, Donna M. Muzny, Harsha Doddapaneni, Jianhong Hu, Eric Boerwinkle, Richard A. Gibbs, Jill A. Rosenfeld, Hong Cui, Fan Xia, Kandamurugu Manickam, Yaping Yang, Eissa A. Faqeih, Ali Al Asmari, Mohammed A. M. Saleh, Ayman W. El-Hattab, James R. Lupski

**Affiliations:** Department of Molecular and Human Genetics, Baylor College of Medicine, Houston, TX 77030 USA; The Baylor-Hopkins Center for Mendelian Genomics, Houston, TX 77030 USA; Human Genome Sequencing Center, Baylor College of Medicine, Houston, TX 77030 USA; Human Genetics Center, University of Texas Health Science Center at Houston, Houston, TX 77030 USA; Exome Laboratory, Baylor Miraca Genetics Laboratories, Houston, TX 77030 USA; Division of Molecular and Human Genetics, Nationwide Children’s Hospital, Columbus, OH 43205 USA; Section of Medical Genetics, Children’s Hospital, King Fahad Medical City, Riyadh, Kingdom of Saudi Arabia; Division of Clinical Genetics and Metabolic Disorders, Department of Pediatrics, Tawam Hospital, Al-Ain, United Arab Emirates; Department of Pediatrics, Baylor College of Medicine, Houston, TX 77030 USA; Department of Pediatrics, Texas Children’s Hospital, Houston, TX 77030 USA

**Keywords:** Whole exome sequencing (WES), Copy Number Variants (CNV), Neurodevelopment, Developmental Delay/Intellectual Disability (DD/ID), *GRM7*

## Abstract

**Background:**

Neurodevelopment is orchestrated by a wide range of genes, and the genetic causes of neurodevelopmental disorders are thus heterogeneous. We applied whole exome sequencing (WES) for molecular diagnosis and *in silico* analysis to identify novel disease gene candidates in a cohort from Saudi Arabia with primarily Mendelian neurologic diseases.

**Methods:**

We performed WES in 31 mostly consanguineous Arab families and analyzed both single nucleotide and copy number variants (CNVs) from WES data. Interaction/expression network and pathway analyses, as well as paralog studies were utilized to investigate potential pathogenicity and disease association of novel candidate genes. Additional cases for candidate genes were identified through the clinical WES database at Baylor Miraca Genetics Laboratories and GeneMatcher.

**Results:**

We found known pathogenic or novel variants in known disease genes with phenotypic expansion in 6 families, disease-associated CNVs in 2 families, and 12 novel disease gene candidates in 11 families, including *KIF5B*, *GRM7*, *FOXP4*, *MLLT1*, and *KDM2B*. Overall, a potential molecular diagnosis was provided by variants in known disease genes in 17 families (54.8 %) and by novel candidate disease genes in an additional 11 families, making the potential molecular diagnostic rate ~90 %.

**Conclusions:**

Molecular diagnostic rate from WES is improved by exome-predicted CNVs. Novel candidate disease gene discovery is facilitated by paralog studies and through the use of informatics tools and available databases to identify additional evidence for pathogenicity.

**Trial registration:**

Not applicable.

**Electronic supplementary material:**

The online version of this article (doi:10.1186/s12920-016-0208-3) contains supplementary material, which is available to authorized users.

## Background

Neurodevelopmental disorders reflect the biology and underlying complexity of nervous system developmental processes, its constituent cellular and anatomical structure, and neurophysiological functions. The human brain cortex consists of nearly 20 billion neurons, each expressing over 80 % of the roughly 25,000 known genes [1] and participating in thousands of synaptic connections [2]. Deleterious gene variants can give rise to a wide range of clinical presentations, which may be divided broadly into either functional (i.e. intellectual disability) or structural (i.e. malformations of cortical development) abnormalities [3]. The study of developmental brain disorders is further challenged by the high degree of genetic heterogeneity observed, even within clinically well-defined disorders, such as pontocerebellar hypoplasia [4]. These observations underscore both the necessity for and power of comprehensive molecular studies in this field [5]. The ultimate goal of these studies is to identify novel neurodevelopmental disease genes and establish potential genotype/phenotype correlations, but the identities of many of these genes remain elusive. Functional studies in animal models and cell lines play a fundamental role in the establishment of genotype/phenotype correlations; however, the need for basic infrastructure and time can be a bottleneck in processing the tremendous amount of data generated by the extensive and widespread use of new genomic tools [6]. The study of Mendelian phenotypes, including neurodevelopmental disorders, provides perhaps the most direct route to identify the link between gene function and resultant phenotype, and a foundation for investigating the underlying biology [7].

In this study, we used whole exome sequencing (WES) to identify potentially deleterious gene variants in a mostly consanguineous cohort from Saudi Arabia with either structural or functional brain abnormalities. CNVs predicted from WES data provide some molecular diagnoses. In addition, investigation of paralogs associated with similar phenotypes also facilitates the identification of novel candidates. Moreover, cross-database gene mining can provide further support for novel candidate disease genes.

## Methods

### Patients

This cohort consists of 31 probands (13 males and 18 females) and their siblings and parents from families with brain malformation and/or developmental delay/intellectual disability. Additional file 1: Table S1 and Additional file 2: Figure S1 provide the pedigree structures and clinical information. All patients were evaluated by clinical geneticists and neurologists.

### Whole exome sequencing (WES) analysis

For the eight multiple-affected families, two members in each family were studied by WES. For the 23 singleton families, four were studied by WES as trios (i.e. affected child plus both parents), and both proband and unaffected twin sibling were studied in 1 family. In the remaining families, only the proband was studied and the DNA samples of other family members were used in segregation analysis. DNA samples from selected individuals were sequenced by WES at the BCM Human Genome Sequencing Center (HGSC) through the Baylor-Hopkins Center for Mendelian Genomics (BHCMG) research initiative. All experiments and analyses were performed as previously described [8], and WES performed in this study had 80X average depth-of-coverage, with 93 % of base positions at a minimum of 20X.

### PCR confirmation for WES data

We used standard PCR to confirm the variants identified from WES and to perform segregation analysis. PCR products were analyzed by Sanger sequencing (DNA Sequencing Core Facility at BCM).

### Absence of heterozygosity (AOH) analysis

Calculated B-allele frequency information was determined from WES as the ratio of variant reads to total reads in WES data and then processed using the Circular Binary Segmentation algorithm implemented in the DNAcopy R bioconductor package [9]. We subtracted 0.5 from the calculated B-allele frequency. Segments with the mean signal > 0.45 and size >1 kb were detected as AOH regions. In the summary statistics in Additional file 1: Table S4, for each proband, we provided the number, the maximum/minimum length, the median length, the mean length, and the total length of potential AOH regions (> = 0.5 Mb).

### CNV analysis from WES data

We used CoNIFER [10], CoNVex (Sanger Centre, unpublished), and HMZDelFinder (https://github.com/BCM-Lupskilab/HMZDelFinder) to predict CNVs from WES data. HMZDelFinder is an in-house-developed algorithm implemented in the R programming language (R Core Team 2014, http://www.R-project.org). WES data were transformed into per-exon read depth, RPKM. Homozygous/hemizygous deletions of exons were defined as meeting three criteria: exons with RPKM <0.5, frequency <0.5 % in the whole cohort, and located within an AOH region (>1 kb), were called together with all the exomes in BHCMG database.

### Evaluation of candidate genes/variants

To assess the candidacy of novel disease genes, we ranked each candidate gene in the cohort from most likely (red) to less (green) according to the following criteria [7]: Genes found in multiple families with similar phenotypes (3 points); Interactors/paralogs of the gene showing overlapping features in human (2 points); Animal studies of the gene or its interactor/paralogs showing overlapping features (2 points); Genomic region associated with the phenotypes (1 point); Loss-of-function variant (2 points); Predicted deleterious by multiple programs (1 point); rare variants in multiple databases (1 point); Expressed in the tissues/organs being affected (1 point).

We further classified variants for all known and novel disease genes using the ACMG/AMP guidelines for variant interpretation in Mendelian disorders (2015) [11] which recommends classification of variant pathogenicity into 5 levels: pathogenic, likely pathogenic, variant of uncertain significance (VUS), likely benign, and benign (Additional file 1: Table S1). As these guidelines provide for additional sub-classification of VUS, we used the term “VUS-favoring likely pathogenic” to indicate variants for which there is evidence of pathogenicity that does not meet criteria for consideration as a likely pathogenic variant. In addition, as the ACMG/AMP guidelines were developed for clinical use and are not designed to address pathogenicity of variants in genes not yet established to be disease-causing, i.e. candidate disease genes in gene discovery efforts, we have categorized all variants in novel candidate disease genes as VUS (Additional file 1: Table S1), while still providing evidence for variant pathogenicity as appropriate.

### Arrays and droplet PCR confirmation for CNVs

Deletion in *GRID2* was confirmed using BCM chromosomal microarray version 10.2 [12]. Droplet digital (dd) PCR for *RPS6KC1* was performed using the QX200™ AutoDG™ Droplet Digital™ PCR System (Bio-Rad) following the manufacturer’s protocols and analyzed in QuantaSoft™. Detailed conditions and primers are listed in Additional file 3: Supplementary Materials and Methods.

## Results

**Fig. 1:**
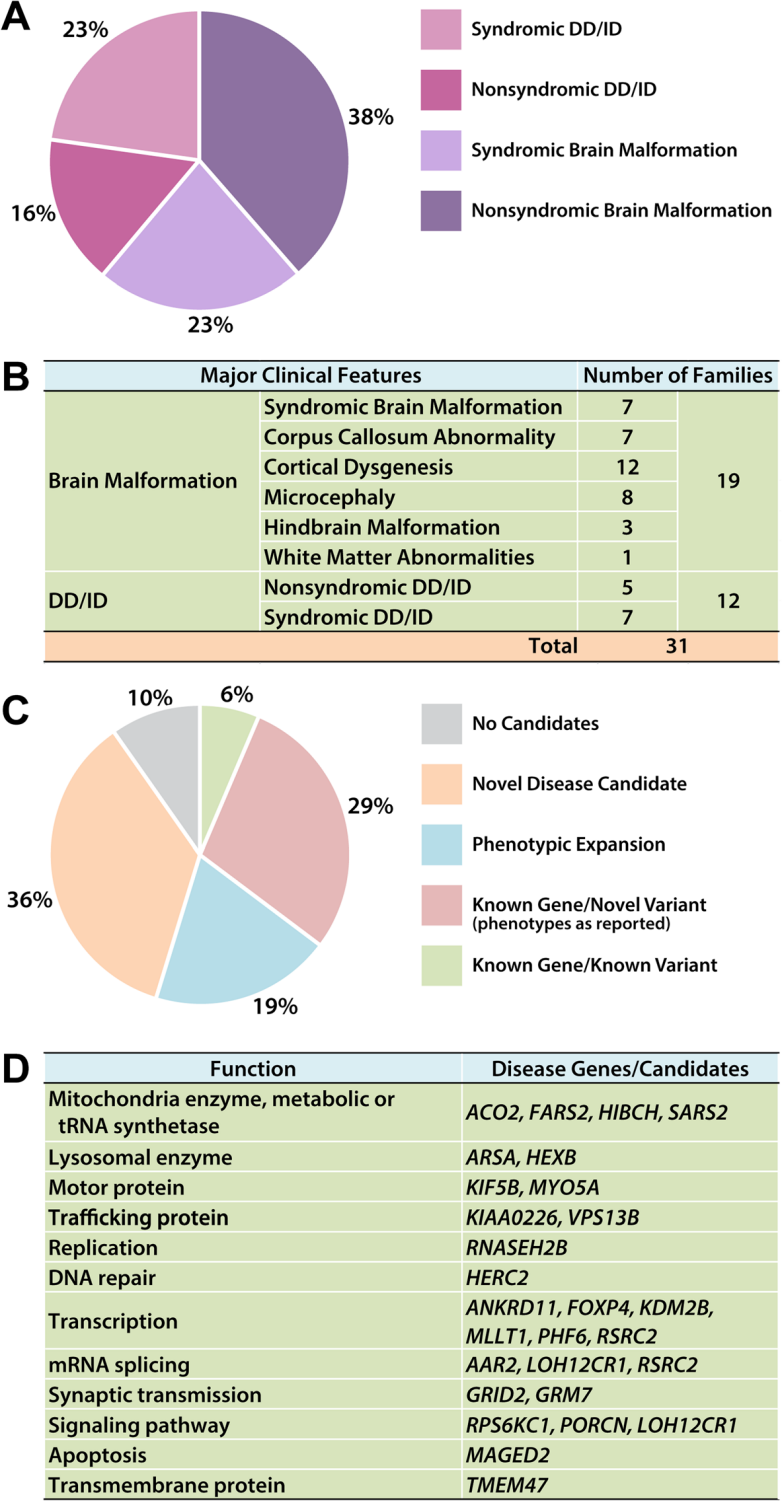
Clinical categories in the cohort and summary of WES findings. **a** Based on the brain structural findings and accompanying features, this cohort is grouped into nonsyndromic brain malformations (38 %), syndromic brain malformations (23 %), nonsyndromic DD/ID (16 %), and syndromic DD/ID (23 %). **b** Based on the brain structural defects, families can be further grouped into corpus callosum abnormalities, cortical dysgenesis, microcephaly, hindbrain malformations, and white matter changes; families may be counted in more than one of these groups. **c** Summary of WES findings (including CNVs): We identified a known variant in a known gene in 2 families (6 %), 10 novel variants in known genes in 9 families presenting reported clinical features (29 %), 7 novel variants in 6 known genes with phenotypic expansion in 6 families (19 %), and 12 novel disease candidates in 11 families (36 %). **d** The disease genes/candidates identified in this study can be grouped into several biological processes, including mitochondrial enzymes (metabolic or tRNA synthetase), lysosomal enzymes, motor proteins, trafficking proteins, DNA replication, DNA repair, transcription, mRNA splicing, synaptic transmission, signaling pathways, apoptosis, and transmembrane proteins

This genomics study includes 31 Arab families exhibiting neurological disease phenotypes, among which 28 were reported to be consanguineous or from the same tribe (Additional file 2: Figure S1). There were 23 (74 %) families with a single affected subject and 8 (26 %) with multiple affected family members. Nineteen (61 %) probands exhibited various structural brain malformations and 12 (39 %) probands presented developmental delay/intellectual disability (DD/ID) without brain malformations (Fig. 1a). We hypothesize that disease causative genes in the first group mainly affect brain development whereas those in the second group mainly affect neuronal functions. The cohort was further clinically assigned into phenotypic subgroups including syndromic brain malformation, corpus callosum abnormalities, cortical dysgenesis, microcephaly, hindbrain malformation, white matter abnormalities, as well as syndromic and non-syndromic DD/ID (Fig. 1b and Additional file 1: Table S2). Individual families could be assigned to multiple subgroups if they had multiple neurodevelopmental phenotypes.

We analyzed both SNVs and CNVs derived from the exome data (Additional file 2: Figure S2). SNVs (including small indels) were computationally filtered using the allele frequency data from different exome databases and bioinformatic predictions of damaging effects on the corresponding protein. In addition to the public exome variant databases, we also searched the variants and genes in our internal BHCMG database with documented clinical features as annotated in PhenoDB [13, 14]. Sanger sequencing of all candidate variants was performed for each proband and all available relatives for confirmation and to assess co-segregation with the phenotype. Only variants that segregated with the phenotype are reported. Additional analyses included an assessment of any previous functional or disease studies of the gene, its orthologs in other model organisms, and its paralogs or known interactors in humans.

We identified rare genetic variants potentially contributing to the phenotypes in ~90 % (28/31) of families studied, including 54.8 % for which a molecular diagnosis involved variants in known disease genes (Fig. 1c-d and Additional file 1: Table S3). Known or suspected consanguinity was reported in 28/31 families and analysis of absence of heterozygosity (AOH) demonstrated one or more regions of AOH > 0.5 Mb in all families with one or more variants in known or candidate disease genes (Additional file 1: Tables S2 and S4). Total personal genome AOH regions ranged from 86.5 Mb to 507.5 Mb in the 28/31 families with historical evidence for consanguinity and from 97.1 Mb to 118.8 Mb in the 3 families in which there was no historical evidence for consanguinity. Most of the variants in potential candidate disease genes that we identified map to the AOH regions and are thus homozygous recessive. In total, we identified 4 *de novo* variants and 1 case with compound heterozygous alleles in candidate genes (Additional file 1: Table S1). One of the *de novo* variants and the one case with compound heterozygous alleles, were found in consanguineous families (Additional file 1: Tables S1 and S2).

Variants in known disease genes included previously described pathogenic variants in 2 families (*FARS2*, *PORCN*) and novel variants in 15 families (*ACO2*, *RNASEH2B*, *SARS2*, *MLC1*, *ARSA*, *HEXB*, *HIBCH*, *HERC2*, *PHF6*, *GRID2*, *MYO5A*, *ANKRD11*, *KIAA0226*, *C12ORF57*, *VPS13B*, *ATP2B3*) (Additional file 1: Table S1), 6 of which are clinically observed to likely represent phenotypic expansion from the previously reported associated traits (Table 1 and Additional file 1: Table S1). Moreover, we propose and provide evidenced-based ranking for 12 novel candidate disease genes (*GRM7*, *FOXP4*, *KIF5B*, *KDM2B*, *MLLT1*, *RPS6KC1*, *CDK20*, *MAGED2*, *TMEM47*, *AAR2*, *LOH12CR1*, *RSRC2*) in 11 families (Table 2 and Additional file 1: Table S1).

**Table 1:** Known disease genes with phenotypic expansion

Family	ID	Gene	Variant	Zyg	Clinical features of patients	MIM	Phenotypic expansion
037	BAB6843	*MYO5A*	NM_000259.3:c.4200C > G:p.Ser1400Arg	Hom	DD, seizures, cerebellar atrophy, nystagmus, dystonia	214450	Lacks classical Griscelli syndrome phenotype of skin hypopigmentation and silver-grey hair
019	BAB6938	*ANKRD11*	NM_013275.5:c.5317G > T:p.E1773*	Het	DD/ID, hypotonia, esotropia, hyperopia, astigmatism, broad nasal bridge, hypertelorism, epicanthal folds, retrognathia, cryptorchidism	148050	Hypotonia, esotropia, hyperopia, astigmatism
022	BAB6787	*KIAA0226*	NM_014687.2:c.1642A > G:p.Thr548Ala	Hom	DD/ID, hypotonia, diffuse cortical hypomyelination, contractures of elbows and knees, nystagmus	615705	Hypotonia, contractures, lacks cerebellar atrophy
			NM_014687.2:c.319G > A:p.Glu107Lys	Hom			
024	BAB6793	*C12ORF57*	NM_138425.3:c.53-2A > G	Hom	DD/ID, ADHD, bilateral hydronephrosis and VUR, atrophic non-functioning left kidney	218340	Vesicoureteric reflux and bilateral hydronephrosis, small non-functioning left kidney
034	BAB6835	*VPS13B*	NM_152564.4:c.1219C > T:p.Gln407*	Hom	DD/ID, ADHD, microcephaly, hypotonia, joint hyperlaxity, unsteady gait, severe dental caries	216550	Autistic behavior, unsteady gait, severe dental caries
026	BAB7039	*ATP2B3*	NM_001001344.2:c.3594G > T:p.Lys1198Asn	Hem	DD/ID, microcephaly, abnormal cortical pattern, hypotonia and muscle atrophy, oropharyngeal dysphagia, dental caries	302500	Microcephaly, abnormal cortical pattern, muscle atrophy, oropharyngeal dysphagia

*DD/ID* developmental delay/intellectual disability, *ADHD* attention deficit hyperactivity disorder, *VUR* Vesicoureteral Reflux, *Hom* homozygous, *Het* heterozygous, *Hem* hemizygous; * stopgain

**Table 2:**
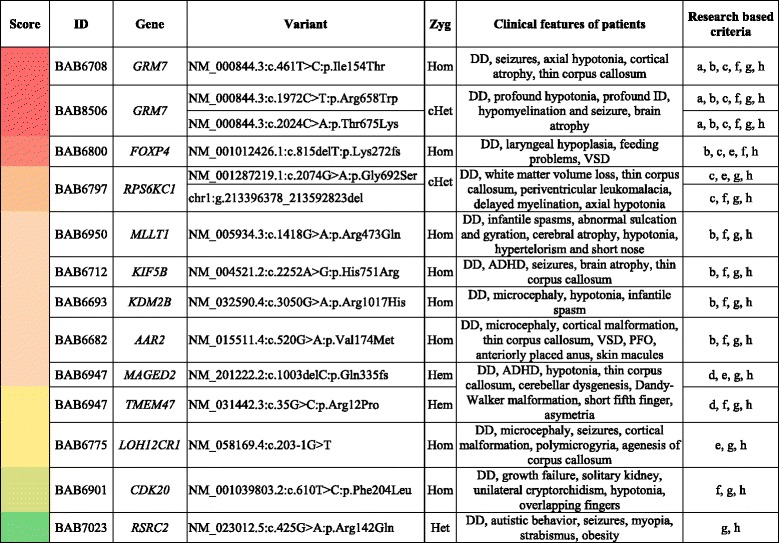
Ranking for the candidate disease genes

The novel candidate genes identified in this cohort were ranked from most likely (red) to less (green) based on the scores using the following criteria: a. Genes found in multiple families with similar phenotypes (3 points); b. Interactors/paralogs of the gene showing overlapping features in human (2 points); c. Animal studies of the gene or its interactor/paralogs showing overlapping features (2 points); d. Genomic region associated with the phenotypes (1 point); e. Loss-of-function variant (2 points); f. Predicted deleterious by multiple programs (1 point); g. Rare variants in multiple databases (1 point); h. Expressed in the tissues/organs being affected (1 point). DD/ID: Developmental Delay/Intellectual Disability; ADHD: attention deficit hyperactivity disorder; PFO: Patent foramen ovale; VSD: Ventricular Septal Defect; VUR: Vesicoureteral Reflux; Hom: homozygous; Het: heterozygous; cHet: compound heterozygous; Hem: hemizygous

### CNVs predicted from WES facilitate establishing a potential etiologic molecular diagnosis

**Fig. 2:**
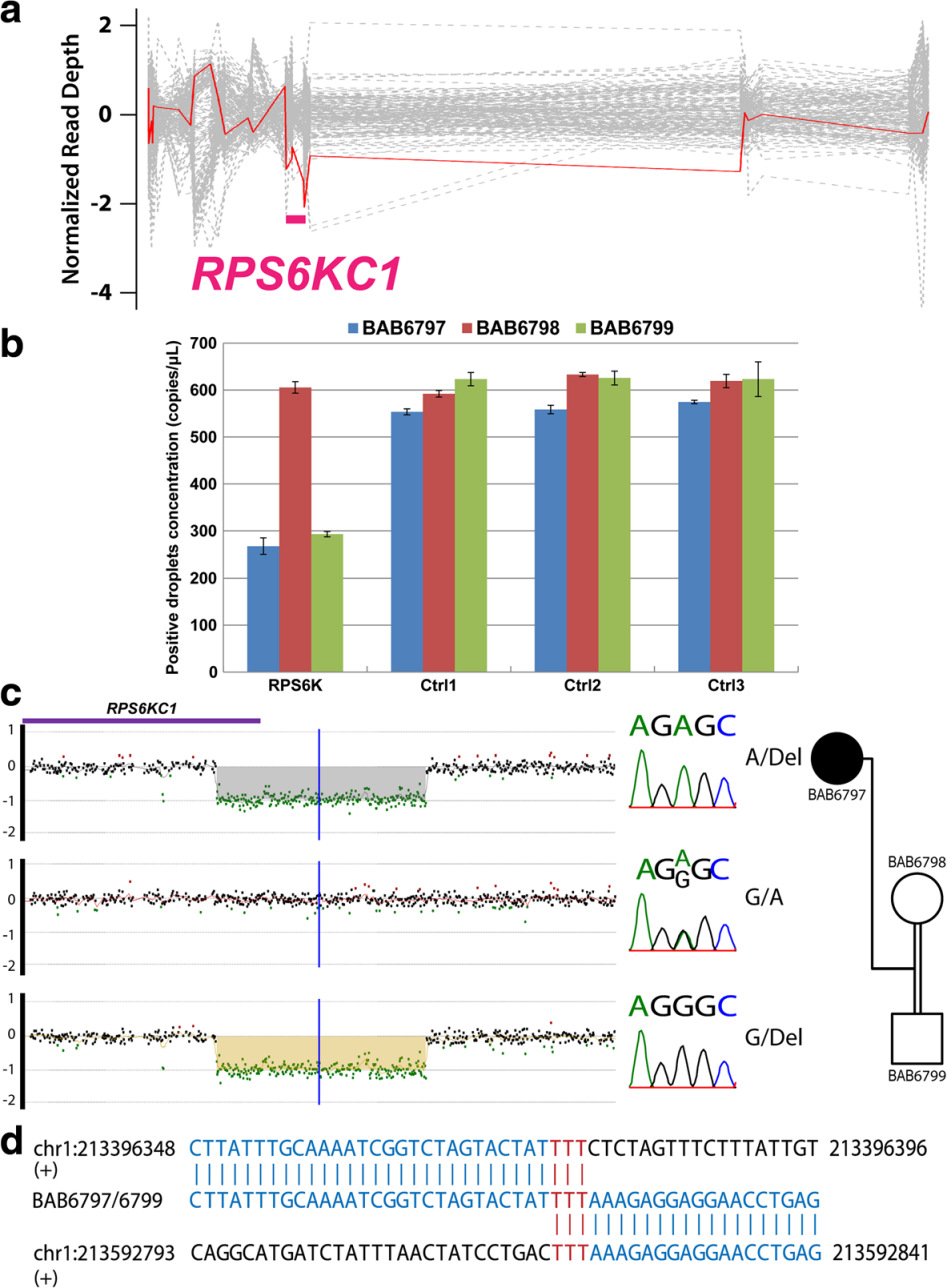
A combination of SNV and CNV in *RPS6KC1* in BAB6797. **a** CoNVex reveals a 42 kb heterozygous deletion (chr1:g.213403839_213445978del) in *RPS6KC1* in BAB6797 (family 025). There is a SNV (NM_001287219.1:c.2074G > A:p.Gly692Ser) uncovered by this deletion. Gray dotted line: the read depth of other samples in the cohort; red line: the read depth for BAB6797; pink line: the predicted deletion region. **b** Droplet Digital PCR (ddPCR) detects heterozygous deletion of *RPS6KC1* in proband BAB6797 and father BAB6799 but not in mother BAB6798; primer pair targeting *RPS6KC1* around chr1:g.213415529[hg19] and three control primer pairs targeting copy-number neutral regions were used to perform ddPCR. Absolute positive droplet concentrations (copies/ul) are plotted from ddPCR results for each primer pair in each sample. Positive droplet concentrations of BAB6797 and BAB6799 for the *RPS6KC1* primer pair (around 300 bp) are approximately half of the value in BAB6798 and the results from all control primers (around 600 bp), indicating heterozygous deletion of the *RPS6KC1* gene in BAB6797 and BAB6799, but not in BAB6798. Corresponding raw data of ddPCR and primer sequences are shown in Table S5 . Ctrl, control. **c** This deletion is also confirmed by a customized array. The combination of SNV and CNV in *RPS6KC1* segregates in the family. **d** Breakpoint junction is mapped to chr1:g.213396378-213592823[hg19] by long-range PCR and Sanger sequencing of the junction

We used CoNIFER [10], CoNVex, and HMZDelFinder to predict potential heterozygous and homozygous CNVs based on exome data. Although many subjects have been pre-screened with karyotyping and array CGH, this bioinformatics approach enables higher resolution evaluation of genomic regions poorly covered by clinical arrays, including homozygous/hemizygous single exon dropout alleles. Among the 31 cases, we found compound heterozygous variant alleles consisting of a SNV and a CNV in a novel candidate gene *RPS6KC1* (Figure 2 and Additional file 1: Table S1) as well as a homozygous intragenic deletion in *GRID2* (Additional file 2: Figure S3).

The combined variants in *RPS6KC1* were identified in the proband (BAB6797) in family 025 with structural brain malformation, including cortical dysgenesis and corpus callosum abnormality (Fig. 2; Additional file 1: Table S1)*.* The SNV in *RPS6KC1* was initially annotated as homozygous (A/A) in the proband; segregation of this variant in the parents demonstrated a heterozygous (G/A) mother and a wild-type (G/G) father, suggesting the possibility of a paternally inherited deletion uncovering the locus of the maternally inherited SNV. Indeed, a heterozygous deletion was predicted by CoNVex (chr1:g.213,403,839-213,445,978[hg19]; 42 kb, including the last six exons of *RPS6KC1*) (Fig. 2a) and CoNIFER (chr1:g.213,341,200-214,171,606[hg19]; 83 kb, including the last seven exons of *RPS6KC1* and the first two exons of the adjacent gene, data not shown). Since the clinical array utilized has suboptimal coverage in this genomic interval, this suspected deletion CNV was confirmed in both father and the proband by droplet digital PCR (Fig. 2b and Additional file 1: Table S5) and a customized high density array CGH (Fig. 2c), and was demonstrated to be 196,446 bp (chr1:g.213396378-213592823[hg19]; including the last 7 exons of this gene) through breakpoint junction PCR (Fig. 2d). *RPS6KC1* mRNA is expressed in the brain (Additional file 2: Figure S4a) and may regulate sphingosine-1 phosphate signaling [15], which is essential for neurogenesis and proper embryonic development [16], suggesting *RPS6KC1* as a candidate disease gene in this family.

The homozygous deletion in *GRID2* was predicted by all three programs in the personal genome WES from proband (BAB6883) in family 041 who presented with hindbrain malformation (Additional file 2: Figure S3a and Additional file 1: Table S1). The deletion predicted by CoNIFER (chr4:g.92007100-94138063[hg19]; 2.1 Mb, data not shown) is much larger than that predicted by HMZDelFinder and CoNVex (chr4:94006145-94032105[hg19]; 25 kb, data not shown), as well as the size experimentally verified by BCM clinical array (chr4:g.93985826-94074965[hg19]; 89 kb) (Additional file 2: Figure S3b) [12]. It was further fine-mapped to chr4:93978239-94078203[hg19] by breakpoint junction PCR (99,965 bp) (Additional file 2: Figure S3c). This homozygous deletion includes exons 3 and 4 of this 15 exon gene. GRID2 is the receptor for excitatory glutamate neurotransmitter in the cerebellum [17] and homozygous deletions, ranging from 37 kb–335 kb in size, were reported to cause cerebellar ataxia and atrophy (MIM#616204).

### Sub-network analysis and cross-database mining provides supporting evidence for candidate genes

In addition to *RPS6KC1*, we identified 11 other potential novel candidate disease genes (Additional file 1: Table S1). The supporting evidence for pathogenicity of these variants is listed in Additional file 1: Table S1 and ranked evidence supporting disease gene candidacy is listed in Table 2. The protein network analysis for individual candidate genes provides potential functional correlation with the phenotypes, such as observed for *CDK20*, *LOH12CR1*, and *RSRC2* (Additional file 1: Table S1). We performed additional bioinformatic analyses of mRNA expression in the brain at different developmental stages, biological functional annotation, and network connectivity based on protein-protein interactions to investigate the novel and known genes as a cohort (Additional file 2: Figure S4 and Figure S5). There is no obvious correlation between the phenotypes and the temporal mRNA expression in the brain. Although the network analysis does not show significant enrichment, which likely reflects the small cohort size as well as heterogeneous phenotypes and genetic causes (Fig. 1d and Additional file 1: Table S1), we observed some sub-networks among a subset of genes, such as *MLLT1* and *KDM2B* (Additional file 2: Figure S5a).

**Fig. 3:**
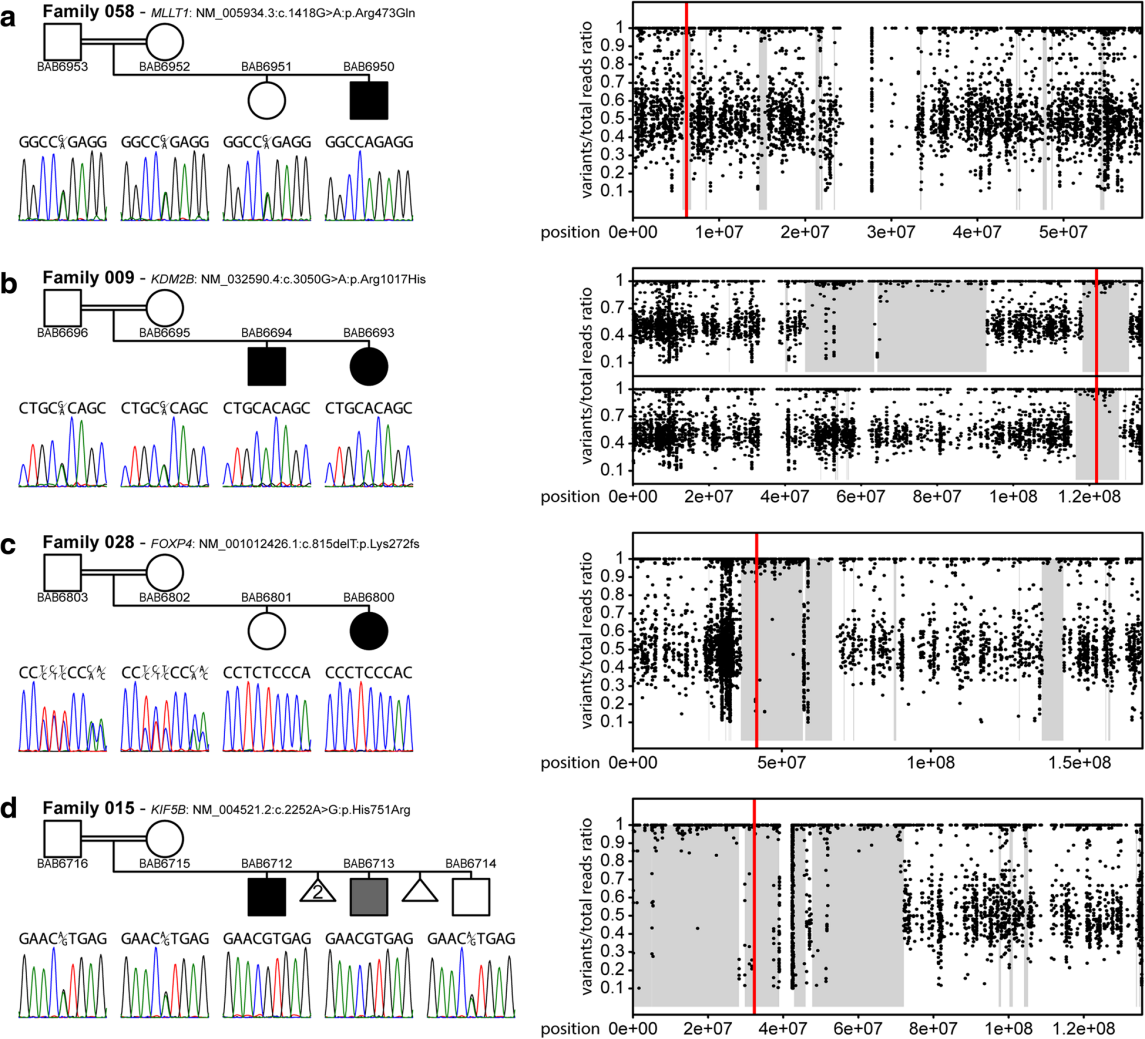
Segregation results and AOH plots of patients with variants in *MLLT1, KDM2B*, *FOXP4*, and *KIF5B.* In each panel, the segregation results are shown to the left and AOH plots on the corresponding chromosomes are shown to the right. **a** In family 058, the proband (BAB6950) has developmental delay, hypotonia, and infantile spasms with diffuse brain atrophy, delay and hypomyelination, as well as malformation of cortical development. A homozygous variant in *MLLT1* (NM_005934.3:c.1418G > A:p.Arg473Gln) is identified and segregates with the phenotypes in the family. **b** In family 009, the proband (BAB6693) has developmental delay, hypotonia, and infantile spasms. A homozygous variant in *KDM2B* (NM_032590.4:c.3050G > A:p.Arg1017His) is identified and segregates with the phenotypes in the family. **c** In family 028, the proband (BAB6800) exhibits developmental delay, laryngeal hypoplasia, and ventricular septal defect (VSD). A homozygous variant in *FOXP4* (NM_001012426.1:c.815delT:p.Lys272fs) is identified and segregates in the family. **d** In family 015, the proband (BAB6712) has developmental delay, seizures, increased reflexes in upper and lower extremities, short stature with mild diffuse brain atrophy, and thinning of the corpus callosum. The younger brother (BAB6713) is mildly affected with speech delay. A homozygous variant in *KIF5B* (NM_004521.2:c.2252A > G:p.His751Arg) is identified and segregates with the phenotypes in the family, present in a homozygous state in both brothers

*MLLT1* is an essential gene during development [18] that co-activates SWI/SNF complexes and regulates histone H3K79 demethylation [19]. The proband (BAB6950) in family 058 with a potentially deleterious homozygous variant in *MLLT1* presents with developmental delay, hypotonia, infantile spasm, and cortical dysgenesis (Additional file 1: Table S1 and Fig. 3a). KDM2B is a histone demethylase for H3K4 and H3K36 [20, 21]. The homozygous variant is identified in two affected siblings (BAB6693 and BAB6694) in family 009 presenting developmental delay, hypotonia, and infantile spasms (Fig. 3b and Additional file 1: Table S1). Variants in the genes involved in chromatin remodeling and histone modification are known to be associated with brain malformation and DD/ID [7].

We previously reported that *Drosophila* genes with multiple human homologs are more likely to be associated with human disorders [8]. We investigated paralogs which have been associated with neurological disorders by adding them into the interaction network and observed sub-network enrichment (Additional file 2: Figure S4b and S5b-d; Additional file 1: Table S6). Paralogs of *FOXP4*, *KIF5B*, and *GRM7*, have been associated with disorders presenting related phenotypes, which provide supporting evidence for these novel candidate disease genes (Fig. 3 and Additional file 1: Table S1).

FOXP4 may play a role in multiple systems affected in the proband (BAB6800) in family 028, such as the larynx and heart [22–24]. Moreover, *FOXP4* and its paralogs, *FOXP1* and *FOXP2*, function together in several developmental processes [25, 26]. Variants in *FOXP1* and *FOXP2* can lead to mental retardation with language impairment (MIM#613670) and Speech-language disorder-1 (MIM#602081), respectively. Genetic abnormalities in *FOXP1* are also associated with congenital heart defects [27]. Therefore, FOXP4 may work together with FOXP1/FOXP2 in brain function and heart development and contribute to the phenotypes observed (Fig. 3c, Additional file 2: Figure S5b, Additional file 1: Table S1).

A homozygous *KIF5B* variant was identified in the proband (BAB6712) and his mildly affected brother (BAB6713) in family 015 (Fig. 3d and Additional file 1: Table S1). *KIF5B* and its paralogs, *KIF5A* and *KIF5C* (Additional file 2: Figure S5c and Additional file 1: Table S6), can transport RNA granules to the neuronal dendrites for protein synthesis and translational silencing [28]. Loss of *KIF5B* is embryonic lethal with severe growth retardation in mice [29]. Moreover, variants in *KIF5A* and *KIF5C* lead to spastic paraplegia (MIM#604187) and cortical dysplasia (MIM#615282), respectively. The severity of the phenotypes in both disorders is variable, which may potentially explain the different severity in the clinical features between the affected siblings. The variability may also be potentially explained by additional unknown genetic variant(s) that exacerbate or suppress clinical severity [30].

**Fig. 4:**
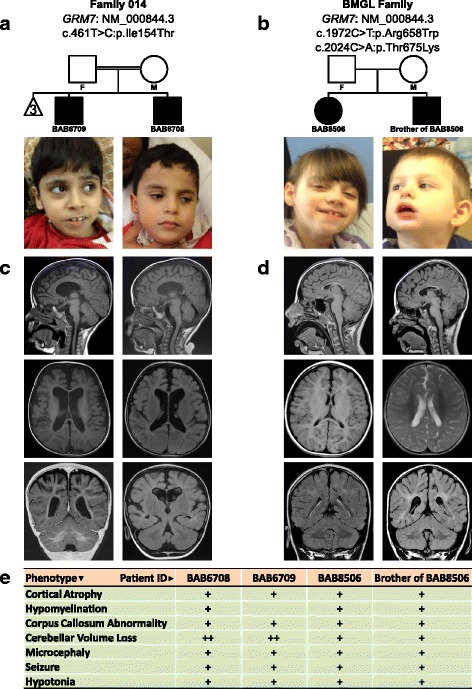
Comparison of the two families carrying *GRM7* variants. **a** Homozygous variant in *GRM7* (NM_000844.3:c.461 T > C:p.Ile154Thr) identified in family 014 with two affected children. **b** Compound heterozygous variants in *GRM7* (NM_000844.3:c.1972C > T:p.Arg658Trp and NM_000844.3:c.2024C > A:p.Thr675Lys) segregates in the family with two affected children identified in the BMGL database. **c** MRI images of BAB6708 and BAB6709. **d** MRI images of BAB8506 and her brother. **e** Clinical features comparison among four patients

*GRM6* and *GRM8,* the paralogs of *GRM7*, have been implicated in neurodevelopmental diseases such as attention deficit hyperactivity disorder (ADHD) and autism spectrum disorders (ASD) [31, 32]. Accordingly, recent genomic studies also revealed an association between *GRM7* and ASD [33, 34]. Another related member, *GRM1* (Additional file 1: Table S6), causes autosomal recessive spinocerebellar ataxia type 13 (MIM#614831) which may include cerebellar atrophy. A recent study indicates GRM7 regulates neurogenesis in early developing mouse brain [35]. Together, these data support *GRM7* as a disease candidate in the two affected siblings (BAB6708 and BAB6709) in family 014 presenting with hypotonia, brain malformation including cortical atrophy, very thin and shortened corpus callosum, and mild cerebellar volume loss (Fig. 4; Additional file 1: Table S1). These examples illustrate how expanding analyses to include paralogs may help identify potential candidate disease genes.

To date, the candidate gene list submitted to GeneMatcher has not resulted in the identification of additional families with phenotypes consistent with those observed in our cohort. However, a similar analysis of candidate genes in clinical WES data from the Baylor-Miraca Genetics Laboratory revealed a family with compound heterozygous *GRM7* variants in two affected siblings presenting similar clinical features, including cortical atrophy, very thin and short corpus callosum, and hypotonia in the affected members of family 014 (Fig. 4). This further supports our findings that *GRM7* is a causative gene for both families.

## Discussion

We performed genomic studies in 31 mostly consanguineous Arab families with either brain malformation or DD/ID and used bioinformatics analyses to identify candidate disease genes and candidate variants likely contributing to the observed clinical neurodevelopmental phenotypes. By considering minor allele frequency, variant properties and predicted effects on protein structure and function, previous functional/disease studies of the gene and its paralogs/interactors, as well as studying both SNVs and CNVs, we identified known disease genes in 55 % and candidate disease genes in 35 % of families (Table 2 and Fig. 1c). The overall potential molecular diagnostic rate considering both known and candidate disease genes is around 90 %. This relatively high rate likely reflects the fact that many neurodevelopmental disease genes have been reported previously and that families included are mostly consanguineous. In this small Arab cohort, there are no shared causative/candidate genes among the families even for the known disease genes. In addition, these genes function in a variety of biological processes, including chromatin modification, transcriptional regulation, signal transduction, intracellular transport, autophagy, mitosis, and apoptosis (Fig. 1d). The diversity of genes found speaks to the heterogeneity of the genetic causes and cellular processes underlying neurological disorders.

### Highlights of a genomic study in a cohort with a high consanguinity rate

We performed WES on two probands in each of the eight families with multiple affected children (Additional file 2: Figure S1). A molecular diagnosis was not achieved in families 006 and 018 since none of the candidate variants assessed segregate with the phenotype among the family members. This may result from shared variants either not being detected or potentially being filtered out by the exome analysis pipeline. Alternatively, an inheritance model that is different than that assumed by pedigree analysis may apply. Although in most of the cases variation at a single locus may explain the clinical features, one must consider the possibility that multiple genetic causes may contribute in a given family and different genes may be involved in different affected family members.

When affected siblings are observed to exhibit variable clinical features in a family, there may still be a shared genetic cause. In family 015 with one severely affected proband and one mildly affected brother, we identified a shared homozygous variant in *KIF5B*. Its paralogs, *KIF5A* and *KIF5C*, are responsible for neurological disorders presenting a variable severity of clinical features, which may also be the case in this family. We cannot exclude the possibility that other gene variants in the proband may contribute to the severity or that other variants in the mildly affected brother may have a protective effect (Additional file 1: Table S1) [30]. Nevertheless, the related functional studies support the molecular diagnosis and *KIF5B* as the major contributor, i.e. the molecular driver variant, for the observed phenotypes (Additional file 1: Table S1).

In family 023, the proband exhibits low activity of arylsulfatase A (ARSA), leading to metachromatic leukodystrophy (MIM#250100). Targeted sequencing for the previously reported *ARSA* variants showed a negative result while WES reveals a novel homozygous *ARSA* variant (Additional file 1: Table S1), likely explaining the low ARSA activity. In family 046, WES was performed on both affected children. Although the analysis pipeline only revealed a homozygous frameshift variant in *ACO2* in the proband (BAB7017) (Additional file 1: Table S1), Sanger confirmation indicated that the affected sibling (BAB7018) also has this homozygous variant. Retrospective re-analysis of the original sequencing files showed that this frameshift in BAB7018 was filtered out in the analysis pipeline due to poor quality metrics. Similarly, frameshift variants in *DVL1*, a recently reported disease gene for Robinow Syndrome (MIM#616331) [36], also evaded detection or were interpreted as missense by the WES analysis pipeline. These examples point out a current challenge in calling indels in WES pipelines. Filtering criteria may improve through utilization of a ‘training set’ of cases with known indels. Alternatively, multiple calling algorithms may be necessary for optimized indel detection.

One challenge in the study of consanguineous families is the presence of multiple rare homozygous variants within extended genomic intervals of AOH that are distributed throughout the genome (Additional file 1: Table S4). This is exemplified by family 001 which is categorized as unsolved because there are multiple candidate variants in different genes that co-segregate with the phenotypes. Other prioritization filters to identify the potential disease contributing variants among rare homozygous variants (Additional file 2: Figure S2 and Table 2) include: i) detecting loss-of-function variants, ii) analyzing potential deleteriousness of missense variants using multiple prediction programs, iii) examining whether the gene is expressed in disease related tissues, iv) assessing available functional studies of the gene or its interactor/paralogs with overlapping phenotypes, and v) finding the gene is a candidate disease gene in multiple families with similar features. In this study, there were 4 *de novo* and 2 compound heterozygous variants found for the potential molecular diagnoses rendered; however, the majority of the disease or candidate variants identified map within AOH regions (Fig. 3 and Additional file 1: Table S4). Therefore, in some families provided with candidate disease genes, there may be additional contributors to the phenotypes (Additional file 1: Table S1).

Estimating the potential inheritance mode based on the pedigree structure before analysis facilitates the speed of analysis. However, all potential inheritance models should be investigated. In family 060, there are eight children with only the youngest affected suggesting a *de novo* event in the proband; therefore trio WES was performed with analysis initially focused on *de novo* variant identification. However, the molecular analysis identified homozygous variants in *RNASEH2B* (NM_001142279.2:c.356A > G:p.Asp119Gly) using a presumed recessive model (Additional file 1: Table S1). Notably, one of the four *de novo* and the compound heterozygous variants are found in consanguineous families (Additional file 1: Tables S1 and S2), indicating the importance of considering all inheritance modes during analysis.

### Expanded phenotypes of the known disease-associated genes and multifunction genes

In family 022, the proband with *KIAA0226* variants exhibits microcephaly without cortical dysgenesis, hindbrain malformation, or corpus callosum abnormality, clinically distinct from the reported spinocerebellar ataxia (MIM#615705). The reported case is a consanguineous Saudi Arabia family with homozygous variants in the same gene, suggesting phenotypic expansion potentially related to intrinsic properties of the identified variants. In family 037 with hindbrain malformation, we identified a homozygous variant in *MYO5A*, which is responsible for Griscelli syndrome (MIM#214450). MYO5A, an actin-based motor protein, regulates many different cellular processes, including melanosome transport in pigment cells [37] and ER transport in Purkinje cells [38]. Different from previously reported truncating variants in Griscelli syndrome, the missense variant in this family seems mainly to affect cerebellar function.

### Further empowering the analysis of the WES data by CNV-detection tools

Predicting CNVs from exome data allows a more complete analysis of possible pathogenic genomic variation, which may result from a variety of mutational mechanisms. The combination of a SNV and a CNV in *RPS6KC1* provides a possible explanation for the proband’s phenotypes in family 025 (Fig. 2, Table 2 and Additional file 1: Table S1). This indicates that by using the read depth information in BAM files and B-allele frequency in VCF files, one can detect intragenic CNV. We anticipate that using next generation sequencing for both CNV and SNV analysis may lead to more molecular diagnoses.

### Paralogs with functional studies and disease association provide additional supportive evidence

Previously, by combining the phenotypic information from a large scale mutagenesis screen in fruit flies with human exome and associated phenotype data from the BHCMG database, we observed that fly genes with multiple human orthologs are more likely to be associated with human disorders [8]. This observation led us to hypothesize that functional or genetic studies of paralogs can facilitate candidate gene identification, as shown in the cases of *FOXP4*, *KIF5B*, and *GRM7*, of which paralogs are closely linked in the interaction network (Additional file 1: Table S6 and Additional file 2: Figure S5). Moreover, utilizing data from clinical sequencing and computational programs for gene matching such as GeneMatcher can provide additional families to support our candidate genes [13].

### WES research study as a building block toward disease gene discovery

In this research-based WES study, we identified several potential candidate disease genes and provide supporting evidence for each gene’s proposed association with disease [7] (Table 2 and Additional file 1: Table S1). We also applied the ACMG variant classification criteria to objectively demonstrate the evidence for pathogenicity for these variants; notably, because the ACMG criteria were designed for the clinical classification of variants in known disease genes, we have classified all variants associated with novel candidate disease genes as VUS [11] (Additional file 1: Table S1). These variant classifications are meant to form a foundation for further study. Nevertheless, this study provides information that may serve as a building block for future disease-gene discovery efforts.

## Conclusions

In summary, by combining traditional WES analysis with WES-derived CNV detection, database mining and bioinformatic analyses of paralog, and interactome studies, we attained a potential molecular diagnosis rate of ~90 % of families in this cohort. We also identified biallelic variants representing recessive disease in a novel disease gene *GRM7* in two families with decreased cerebral and cerebellar volume. Our data suggest that this combined strategy for WES analysis can provide a high potential molecular diagnostic rate in this genetically heterogeneous cohort exhibiting neurologic disease.

## Abbreviations

ADHD, attention deficit hyperactivity disorder; AOH, absence of heterozygosity; ASD, autism spectrum disorders; BHCMG, Baylor-Hopkins Center for Mendelian Genomics; CNV, copy number variant; DD/ID, developmental delay/intellectual disability; ddPCR, droplet digital PCR; HGSC, human genome sequencing center; PFO, patent foramen ovale; VSD, ventricular septal defect; VUR, vesicoureteral reflux; WES, whole exome sequencing

## Additional files

Additional file 1: Table S1. Summary of clinical features and disease/candidate variants identified. The major clinical features and the disease/candidate variants as well as the prediction scores and classifications for damaging effect of the variants are listed. For Polyphen2 (Pph2), D for probably damaging, P for possibly damaging, and B for benign. For LRT, D for deleterious, N for neutral, and U for unknown. For MutationTaster (MT), A for disease causing automatic, D for disease causing, N for polymorphism, and P for polymorphism automatic. Moreover, this table includes the ranking and ACMG criteria for each gene, the supporting evidence and the discussion of other variants, as well as the frequency/number of the variants in our internal CMG database, Atherosclerosis Risk in Communities Study (ARIC), ExAC database, Thousand Genome project, and NHLBI GO Exome Sequencing Project (ESP). pLI: probability of loss-of-function (LoF) intolerance. **Table S2.** Categorization of families based on major clinical features. Y: the family has this clinical feature; N: the family does not have this clinical feature. Families with brain malformations were not counted in the ID/DD groups, even if this feature was present. Percentages of families with each feature are shown at the bottom of the table. **Table S3.** Categorization of disease genes/candidates by major clinical features. **Table S4.** AOH metrics for the probands carrying known or candidate disease genes. **Table S5.** Raw data of ddPCR in *RPS6KC1* in family 025. **Table S6.** Homologs of disease genes/candidates between human and fruit fly. The HCOP website (http://www.genenames.org/cgi-bin/hcop) was used to identify the fly homologs of the identified disease/candidate genes, listed in the upper panel. These fly homologs are then used to search for additional human homologs to find paralogs of the original human genes, as shown in the bottom part of the list. (XLS 165 kb) Additional file 2: Figure S1. Thirty one Arab family pedigrees in this study. Family numbers are given as numeric series and individual IDs as BAB series. They are arranged into: known variant in known gene (green), novel variant in known gene (light red), phenotypic expansion (blue), novel disease candidates (light orange), and unsolved cases (gray). Gray shading indicates a minor phenotype. **Figure S2.** Workflow of WES analysis. The SNVs predicted to have (probably) damaging effects are filtered with the allele frequency data from Atherosclerosis Risk in Communities Study (ARIC) (http://drupal.cscc.unc.edu/aric/), NHLBI GO Exome Sequencing Project (ESP), Seattle, WA (http://evs.gs.washington.edu/EVS/), 1000 Genomes Project (http://www.1000genomes.org), and Exome Aggregation Consortium (ExAC) Cambridge, MA (http://exac.broadinstitute.org) [Oct 2014] and Baylor-Hopkins Center for Mendelian Genomics (BHCMG) database with more than 5,000 exomes. WES data are also used to predict CNVs. The information of the paralogs of candidate genes provides supporting evidence for the findings. Moreover, cross-database gene mining identifies additional cases. **Figure S3.** CNV prediction identifies a homozygous deletion in *GRID2* in BAB6883. a HMZDelFinder reveals a homozygous deletion in *GRID2* (chr4:g.94006145_94032105del). The lower threshold for RPKM is 0.5 and upper threshold is 1. b This deletion is confirmed by clinical array (chr4:g.93,985,826_94,074,965del) and segregates with phenotypes in the family. c Breakpoint junction maps to chr4:g.93,978,239_94,078,203del. **Figure S4.** Network analysis of known and candidate genes. a Temporal RNA expression in brain. b The protein-protein interaction network: Interaction STRING scores are displayed in an upper triangular heatmap. Low and high values are represented in blue and red, respectively. **Figure S5.** Protein interaction network of the candidate genes. a KDM2B and MLLT1 are in the chromatin remodeling sub-network. b FOXP4 and its paralogs. c KIF5B and its paralogs. d GRM7 and its related members. (ZIP 4859 kb) Additional file 3: Suplementary Materials and Methods. (DOCX 16 kb)
