# Supplementary figures and images for: Exome sequencing in mostly consanguineous Arab families with neurologic disease provides a high potential molecular diagnosis rate
Source: BMC Med Genomics. 2016 Jul 19;9:42. doi: 10.1186/s12920-016-0208-3 (PMC4950750; doi:10.1186/s12920-016-0208-3)

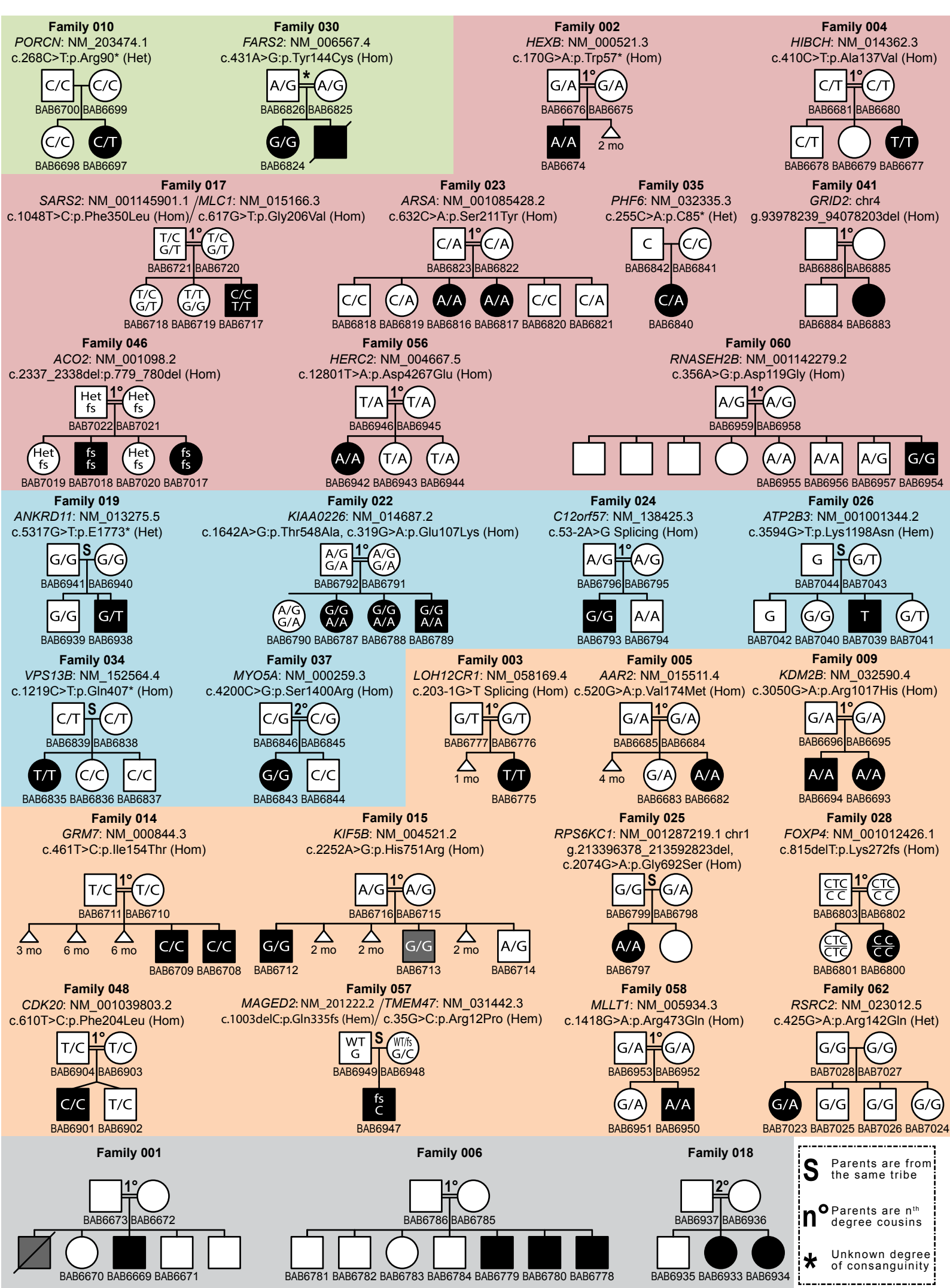

Figure S1

Supplement: Additional file 2: Figure S1. — Thirty one Arab family pedigrees in this study. Family numbers are given as numeric series and individual IDs as BAB series. They are arranged into: known variant in known gene (green), novel variant in known gene (light red), phenotypic expansion (blue), novel disease candidates (light orange), and unsolved cases (gray). Gray shading indicates a minor phenotype. Figure S2. Workflow of WES analysis. The SNVs predicted to have (probably) damaging effects are filtered with the allele frequency data from Atherosclerosis Risk in Communities Study (ARIC) (http://drupal.cscc.unc.edu/aric/), NHLBI GO Exome Sequencing Project (ESP), Seattle, WA (http://evs.gs.washington.edu/EVS/), 1000 Genomes Project (http://www.1000genomes.org), and Exome Aggregation Consortium (ExAC) Cambridge, MA (http://exac.broadinstitute.org) [Oct 2014] and Baylor-Hopkins Center for Mendelian Genomics (BHCMG) database with more than 5,000 exomes. WES data are also used to predict CNVs. The information of the paralogs of candidate genes provides supporting evidence for the findings. Moreover, cross-database gene mining identifies additional cases. Figure S3. CNV prediction identifies a homozygous deletion in GRID2 in BAB6883. a HMZDelFinder reveals a homozygous deletion in GRID2 (chr4:g.94006145_94032105del). The lower threshold for RPKM is 0.5 and upper threshold is 1. b This deletion is confirmed by clinical array (chr4:g.93,985,826_94,074,965del) and segregates with phenotypes in the family. c Breakpoint junction maps to chr4:g.93,978,239_94,078,203del. Figure S4. Network analysis of known and candidate genes. a Temporal RNA expression in brain. b The protein-protein interaction network: Interaction STRING scores are displayed in an upper triangular heatmap. Low and high values are represented in blue and red, respectively. Figure S5. Protein interaction network of the candidate genes. a KDM2B and MLLT1 are in the chromatin remodeling sub-network. b FOXP4 and its paralogs. c KIF5B and its paralogs. [file 12920_2016_208_MOESM2_ESM.zip › Figure S1_A4formatR2.pdf]

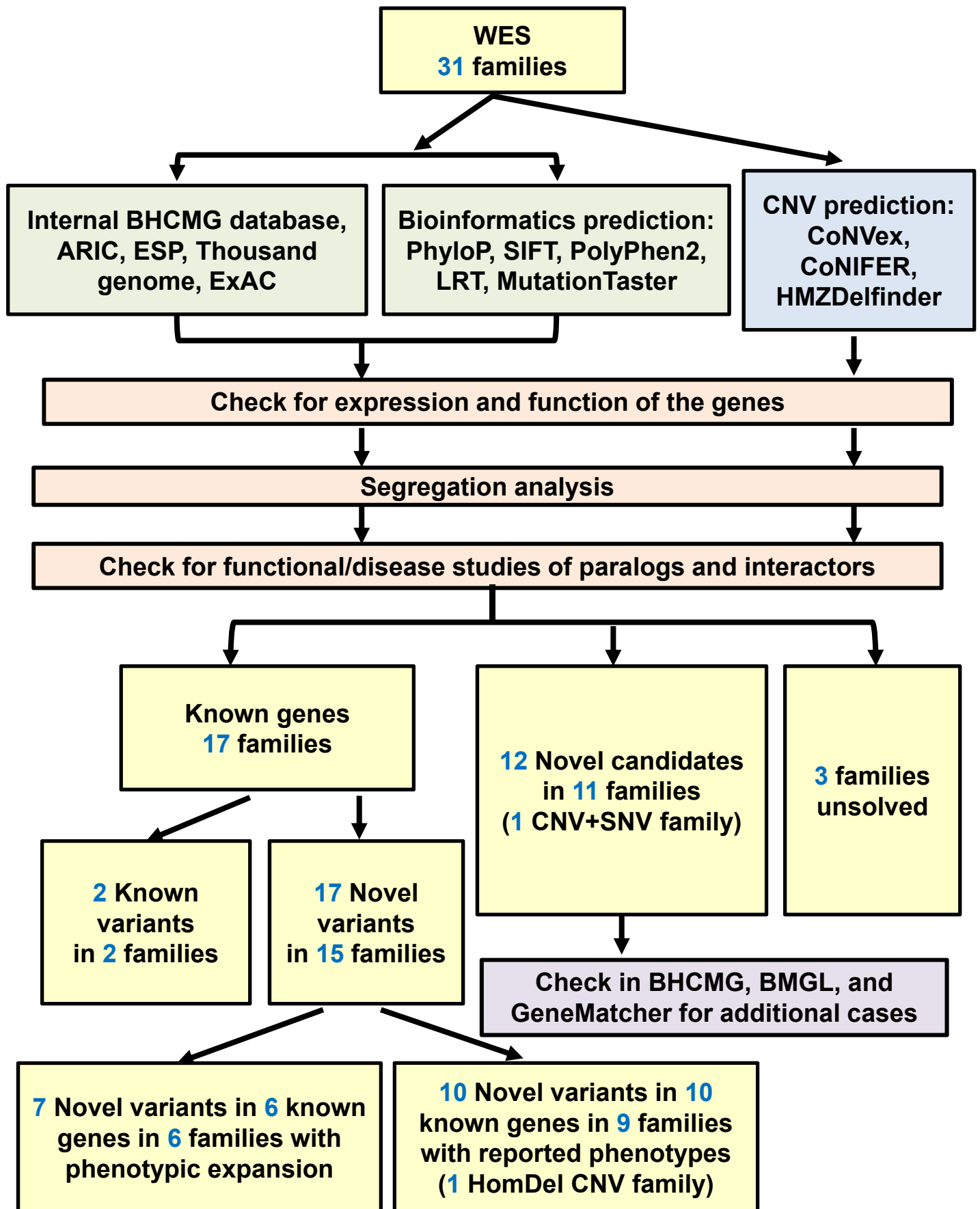

**Figure S2**

Supplement: Additional file 2: Figure S1. — Thirty one Arab family pedigrees in this study. Family numbers are given as numeric series and individual IDs as BAB series. They are arranged into: known variant in known gene (green), novel variant in known gene (light red), phenotypic expansion (blue), novel disease candidates (light orange), and unsolved cases (gray). Gray shading indicates a minor phenotype. Figure S2. Workflow of WES analysis. The SNVs predicted to have (probably) damaging effects are filtered with the allele frequency data from Atherosclerosis Risk in Communities Study (ARIC) (http://drupal.cscc.unc.edu/aric/), NHLBI GO Exome Sequencing Project (ESP), Seattle, WA (http://evs.gs.washington.edu/EVS/), 1000 Genomes Project (http://www.1000genomes.org), and Exome Aggregation Consortium (ExAC) Cambridge, MA (http://exac.broadinstitute.org) [Oct 2014] and Baylor-Hopkins Center for Mendelian Genomics (BHCMG) database with more than 5,000 exomes. WES data are also used to predict CNVs. The information of the paralogs of candidate genes provides supporting evidence for the findings. Moreover, cross-database gene mining identifies additional cases. Figure S3. CNV prediction identifies a homozygous deletion in GRID2 in BAB6883. a HMZDelFinder reveals a homozygous deletion in GRID2 (chr4:g.94006145_94032105del). The lower threshold for RPKM is 0.5 and upper threshold is 1. b This deletion is confirmed by clinical array (chr4:g.93,985,826_94,074,965del) and segregates with phenotypes in the family. c Breakpoint junction maps to chr4:g.93,978,239_94,078,203del. Figure S4. Network analysis of known and candidate genes. a Temporal RNA expression in brain. b The protein-protein interaction network: Interaction STRING scores are displayed in an upper triangular heatmap. Low and high values are represented in blue and red, respectively. Figure S5. Protein interaction network of the candidate genes. a KDM2B and MLLT1 are in the chromatin remodeling sub-network. b FOXP4 and its paralogs. c KIF5B and its paralogs. [file 12920_2016_208_MOESM2_ESM.zip › Figure S2_A4formatR2.pdf]

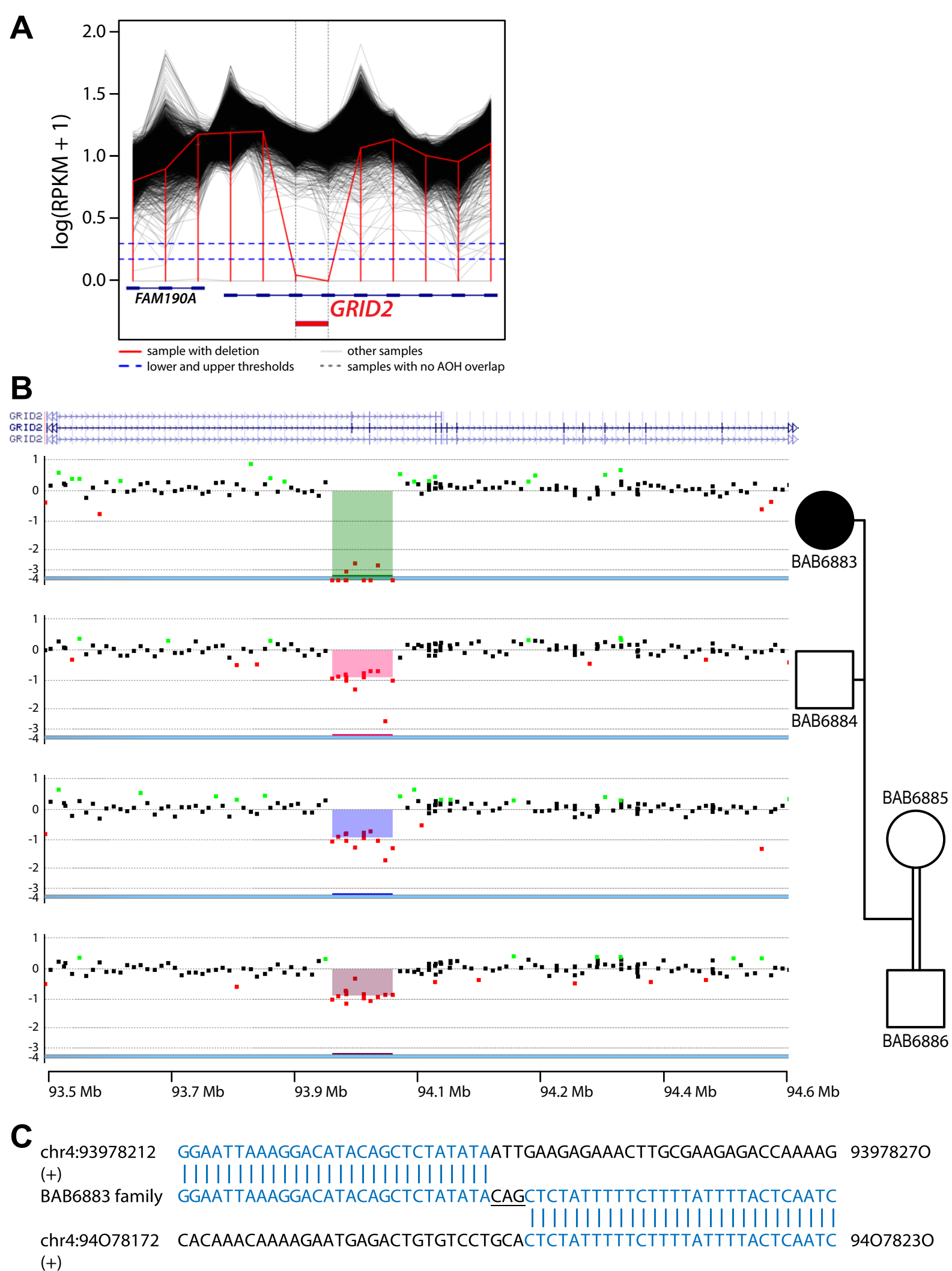

Figure S3

Supplement: Additional file 2: Figure S1. — Thirty one Arab family pedigrees in this study. Family numbers are given as numeric series and individual IDs as BAB series. They are arranged into: known variant in known gene (green), novel variant in known gene (light red), phenotypic expansion (blue), novel disease candidates (light orange), and unsolved cases (gray). Gray shading indicates a minor phenotype. Figure S2. Workflow of WES analysis. The SNVs predicted to have (probably) damaging effects are filtered with the allele frequency data from Atherosclerosis Risk in Communities Study (ARIC) (http://drupal.cscc.unc.edu/aric/), NHLBI GO Exome Sequencing Project (ESP), Seattle, WA (http://evs.gs.washington.edu/EVS/), 1000 Genomes Project (http://www.1000genomes.org), and Exome Aggregation Consortium (ExAC) Cambridge, MA (http://exac.broadinstitute.org) [Oct 2014] and Baylor-Hopkins Center for Mendelian Genomics (BHCMG) database with more than 5,000 exomes. WES data are also used to predict CNVs. The information of the paralogs of candidate genes provides supporting evidence for the findings. Moreover, cross-database gene mining identifies additional cases. Figure S3. CNV prediction identifies a homozygous deletion in GRID2 in BAB6883. a HMZDelFinder reveals a homozygous deletion in GRID2 (chr4:g.94006145_94032105del). The lower threshold for RPKM is 0.5 and upper threshold is 1. b This deletion is confirmed by clinical array (chr4:g.93,985,826_94,074,965del) and segregates with phenotypes in the family. c Breakpoint junction maps to chr4:g.93,978,239_94,078,203del. Figure S4. Network analysis of known and candidate genes. a Temporal RNA expression in brain. b The protein-protein interaction network: Interaction STRING scores are displayed in an upper triangular heatmap. Low and high values are represented in blue and red, respectively. Figure S5. Protein interaction network of the candidate genes. a KDM2B and MLLT1 are in the chromatin remodeling sub-network. b FOXP4 and its paralogs. c KIF5B and its paralogs. [file 12920_2016_208_MOESM2_ESM.zip › Figure S3_A4formatR2.pdf]

A

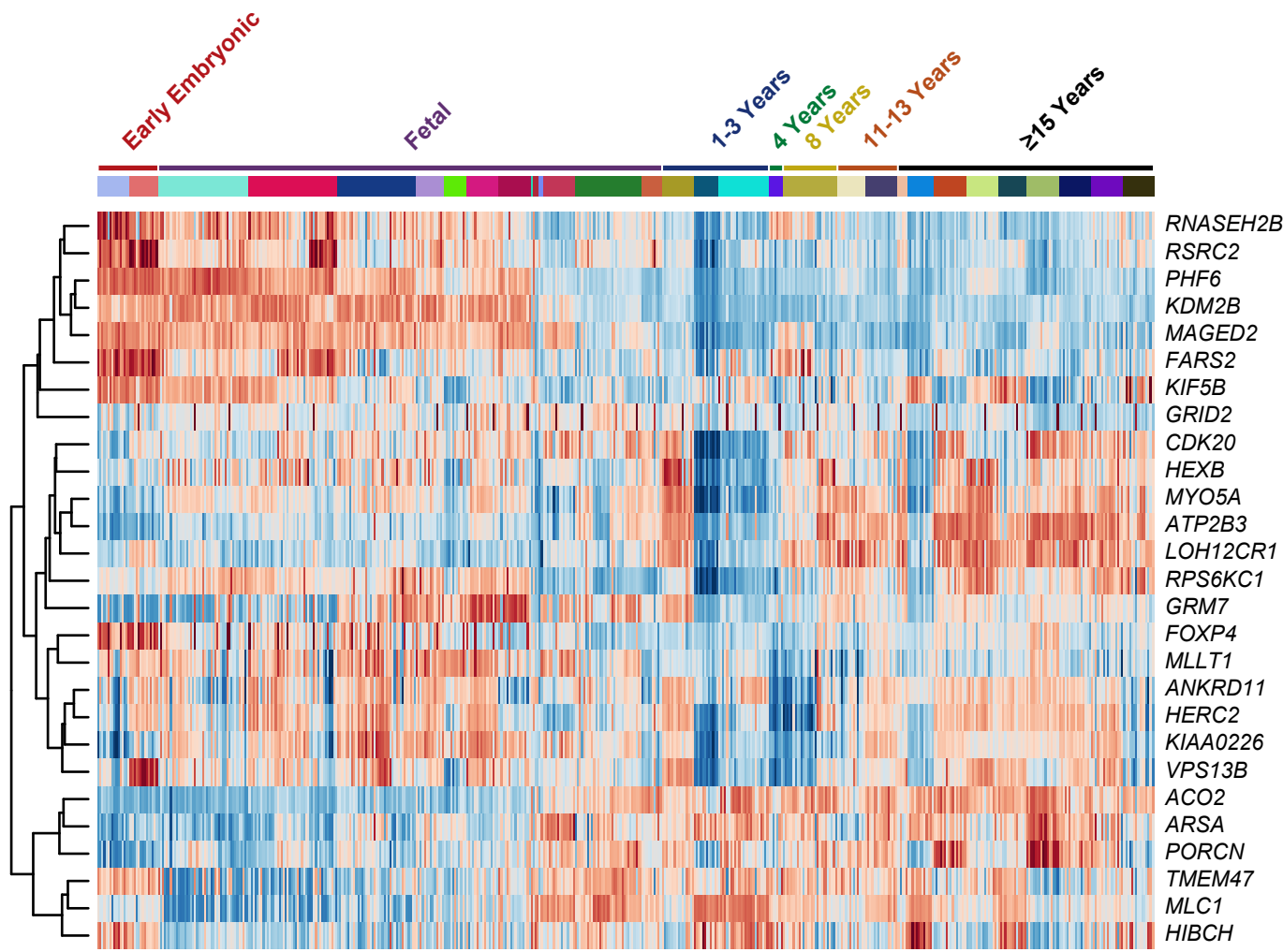

B

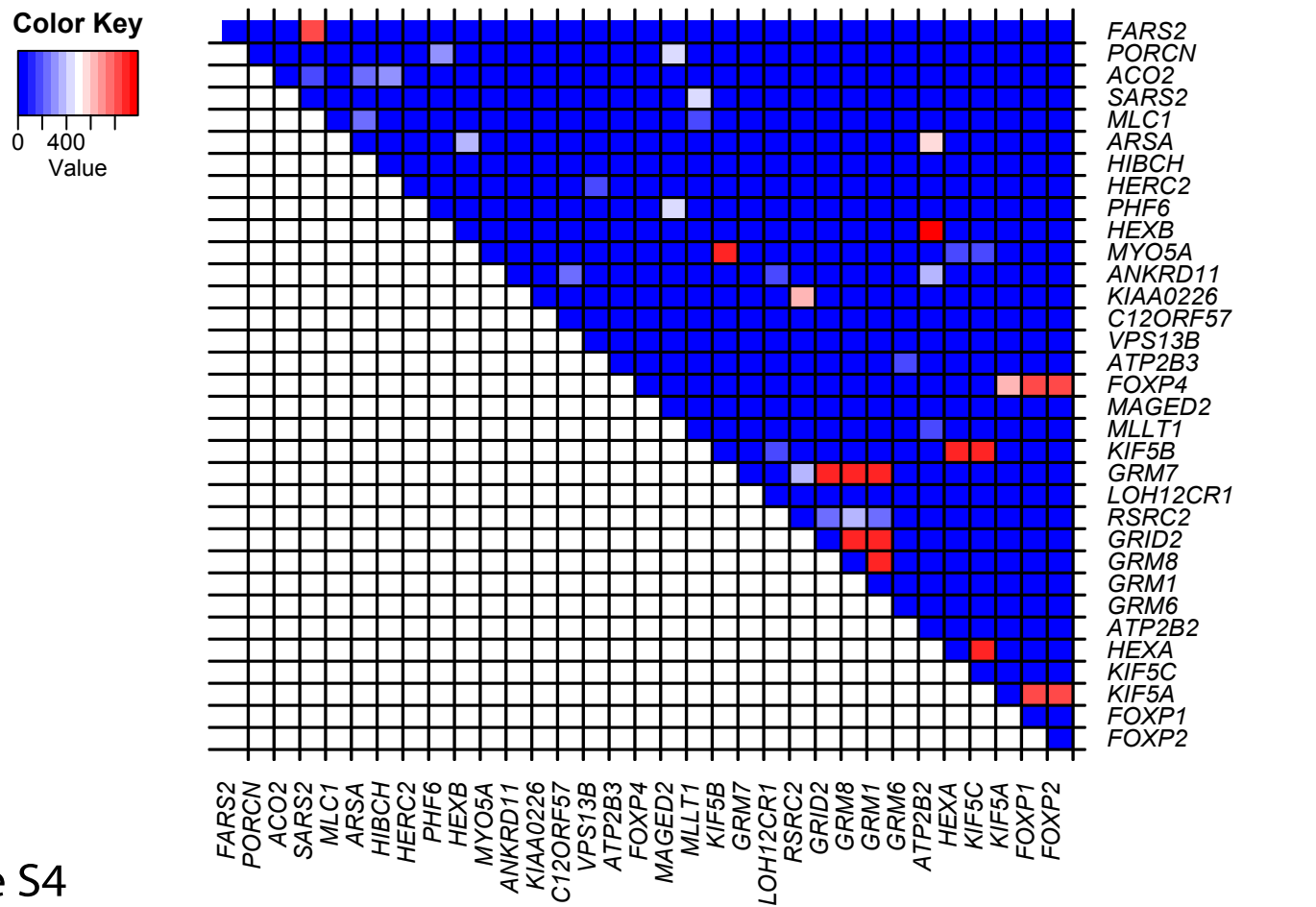

Figure S4

Supplement: Additional file 2: Figure S1. — Thirty one Arab family pedigrees in this study. Family numbers are given as numeric series and individual IDs as BAB series. They are arranged into: known variant in known gene (green), novel variant in known gene (light red), phenotypic expansion (blue), novel disease candidates (light orange), and unsolved cases (gray). Gray shading indicates a minor phenotype. Figure S2. Workflow of WES analysis. The SNVs predicted to have (probably) damaging effects are filtered with the allele frequency data from Atherosclerosis Risk in Communities Study (ARIC) (http://drupal.cscc.unc.edu/aric/), NHLBI GO Exome Sequencing Project (ESP), Seattle, WA (http://evs.gs.washington.edu/EVS/), 1000 Genomes Project (http://www.1000genomes.org), and Exome Aggregation Consortium (ExAC) Cambridge, MA (http://exac.broadinstitute.org) [Oct 2014] and Baylor-Hopkins Center for Mendelian Genomics (BHCMG) database with more than 5,000 exomes. WES data are also used to predict CNVs. The information of the paralogs of candidate genes provides supporting evidence for the findings. Moreover, cross-database gene mining identifies additional cases. Figure S3. CNV prediction identifies a homozygous deletion in GRID2 in BAB6883. a HMZDelFinder reveals a homozygous deletion in GRID2 (chr4:g.94006145_94032105del). The lower threshold for RPKM is 0.5 and upper threshold is 1. b This deletion is confirmed by clinical array (chr4:g.93,985,826_94,074,965del) and segregates with phenotypes in the family. c Breakpoint junction maps to chr4:g.93,978,239_94,078,203del. Figure S4. Network analysis of known and candidate genes. a Temporal RNA expression in brain. b The protein-protein interaction network: Interaction STRING scores are displayed in an upper triangular heatmap. Low and high values are represented in blue and red, respectively. Figure S5. Protein interaction network of the candidate genes. a KDM2B and MLLT1 are in the chromatin remodeling sub-network. b FOXP4 and its paralogs. c KIF5B and its paralogs. [file 12920_2016_208_MOESM2_ESM.zip › Figure S4_A4formatR2.pdf]

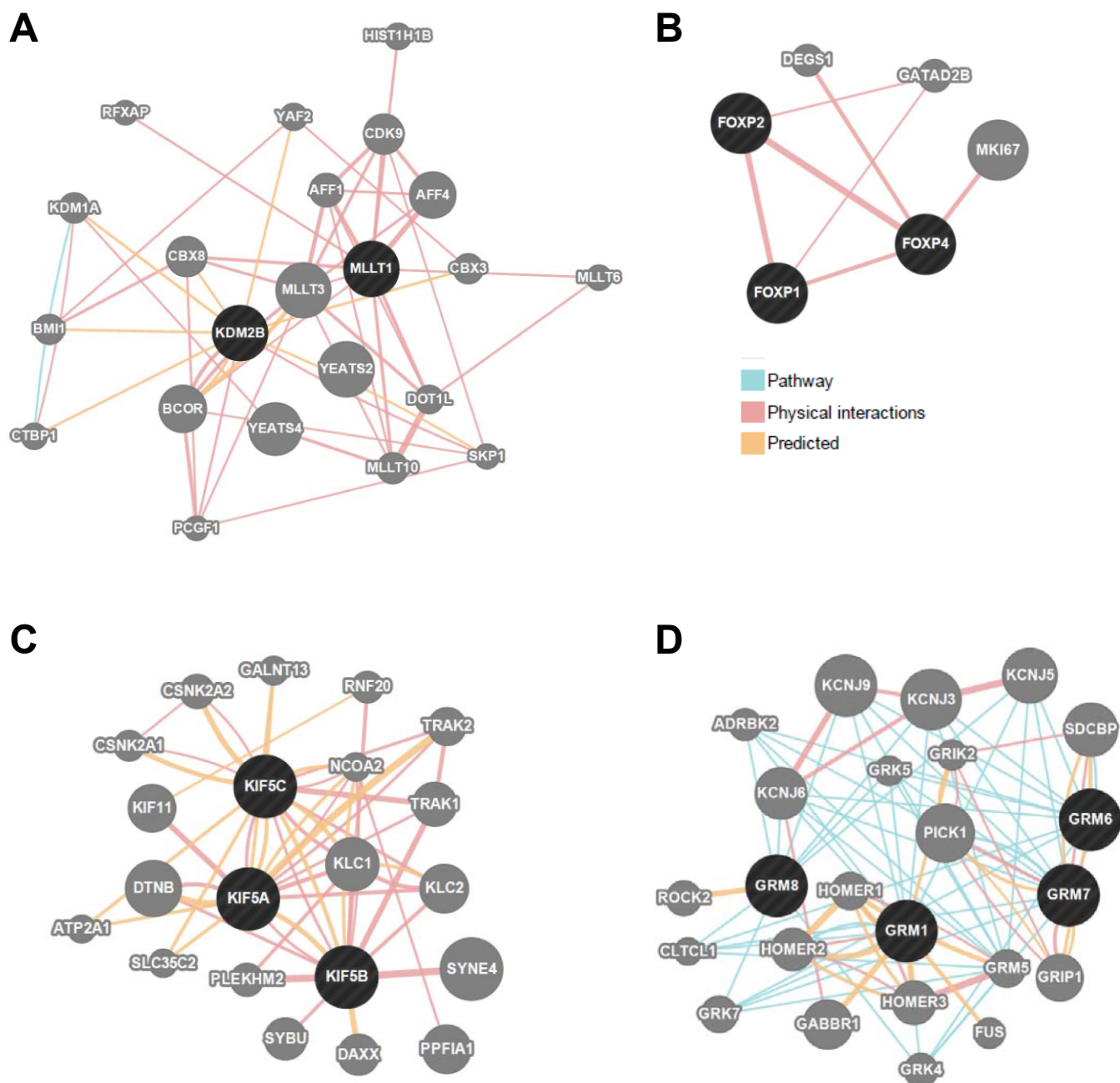

**Figure S5**

Supplement: Additional file 2: Figure S1. — Thirty one Arab family pedigrees in this study. Family numbers are given as numeric series and individual IDs as BAB series. They are arranged into: known variant in known gene (green), novel variant in known gene (light red), phenotypic expansion (blue), novel disease candidates (light orange), and unsolved cases (gray). Gray shading indicates a minor phenotype. Figure S2. Workflow of WES analysis. The SNVs predicted to have (probably) damaging effects are filtered with the allele frequency data from Atherosclerosis Risk in Communities Study (ARIC) (http://drupal.cscc.unc.edu/aric/), NHLBI GO Exome Sequencing Project (ESP), Seattle, WA (http://evs.gs.washington.edu/EVS/), 1000 Genomes Project (http://www.1000genomes.org), and Exome Aggregation Consortium (ExAC) Cambridge, MA (http://exac.broadinstitute.org) [Oct 2014] and Baylor-Hopkins Center for Mendelian Genomics (BHCMG) database with more than 5,000 exomes. WES data are also used to predict CNVs. The information of the paralogs of candidate genes provides supporting evidence for the findings. Moreover, cross-database gene mining identifies additional cases. Figure S3. CNV prediction identifies a homozygous deletion in GRID2 in BAB6883. a HMZDelFinder reveals a homozygous deletion in GRID2 (chr4:g.94006145_94032105del). The lower threshold for RPKM is 0.5 and upper threshold is 1. b This deletion is confirmed by clinical array (chr4:g.93,985,826_94,074,965del) and segregates with phenotypes in the family. c Breakpoint junction maps to chr4:g.93,978,239_94,078,203del. Figure S4. Network analysis of known and candidate genes. a Temporal RNA expression in brain. b The protein-protein interaction network: Interaction STRING scores are displayed in an upper triangular heatmap. Low and high values are represented in blue and red, respectively. Figure S5. Protein interaction network of the candidate genes. a KDM2B and MLLT1 are in the chromatin remodeling sub-network. b FOXP4 and its paralogs. c KIF5B and its paralogs. [file 12920_2016_208_MOESM2_ESM.zip › Figure S5_A4formatR2.pdf]
