# Supplementary material for: Exome sequencing in mostly consanguineous Arab families with neurologic disease provides a high potential molecular diagnosis rate
Source: BMC Med Genomics. 2016 Jul 19;9:42. doi: 10.1186/s12920-016-0208-3 (PMC4950750; doi:10.1186/s12920-016-0208-3)
Supplement: Additional file 3: — Suplementary Materials and Methods. (DOCX 16 kb) [file 12920_2016_208_MOESM3_ESM.docx]

**Suplementary Materials and Methods**

**ddPCR detailed protocol**

ddPCR was performed using the QX200™ AutoDG™ Droplet Digital™ PCR System from Bio-Rad following manufacturer’s protocols. Briefly, a 20 ul mixture was set up for each PCR reaction, containing 10ul of 2x Q200 ddPCR EvaGreen Supermix, 0.25ul of each primer (10uM) and 20ng of genomic DNA. Reaction mixture was subjected to automatic droplet generation, followed by PCR reaction and droplet reading. Cycling conditions for PCR are as the following: 5 mins at 95ºC, 40 cycles of 30 seconds (sec) at 95ºC / 1 min at 65ºC / 1 min at 72ºC, 5 mins at 4ºC, 5 mins at 90ºC and finally infinite hold at 4ºC. Ramp rate was set for 2ºC per sec for all steps. Data was analyzed using QuantaSoft^TM^ Software from Bio-Rad, and concentrations of positive droplets (number of positive droplets per ul of reaction) were obtained for each PCR reaction.

| Primer | Sequence (5'-3') |
| --- | --- |
| RPS6K-ddF | GGGTGAAAGTGGTTTAGTGCTAGAAGGA |
| RPS6K-ddR | TGTAAAGCATCAAGGGCTACCACCA |
| Ctrl1-ddF | GACTCCATTTCTGTGGGTGTTGGTT |
| Ctrl1-ddR | GTTGCCCCTGGATTAACCCTCTTTT |
| Ctrl2-ddF | CGCTTGTAAGCAACCATGAGATGTG |
| Ctrl2-ddR | TGCCTACTGTGGTTGGATGTGATGT |
| Ctrl3-ddF | CCGCACTCAATAAAGGAATGGACAC |
| Ctrl3-ddR | CTTGGGGCATAAGGTAGGGACTCAG |

**Expression Heatmap Generation**

The normalized expression values for all of our candidate genes including known and novel genes were retrieved from <http://www.brain-map.org> for 13 different stages of brain development over 16 substructures. These genes were hierarchically clustered based on their normalized expression values, using the heat plot function in the made4 Bioconductor package.

**Network Analysis**

In Figure S4, the combined interaction score between every possible mutated gene pair was interrogated, using the STRINGdb Bioconductor package. The interaction scores were represented in a heatmap.2 function in R. In Figure S5, the interaction network is obtained using GeneMANIA.
